# Analectic

**Published:** 1829-02

**Authors:** 


### MISCELLANEOUS INTELLIGENCE.

### I.—Analectic.

1. *On the Valves of the Pulmonary Veins*, by Professor Mayer. 'In all systematic works on anatomy, we find it asserted that the pulmonary veins have no valves. It is unnecessary to prove this by multiplied citations—Waller among the ancients, and Meckel as the most modern writer, will suffice. The former says, in his Elementa Physiologiæ, t. i. p. 145, "Sed etiam vera pulmonalis absque valvulis est;"—and Meckel, in his Human Anatomy, vol. iii. p. 368, remarks that the pulmonary veins are usually without valves, with some very rare exceptions.

Professor Mayer's attention was first called to the valves in these vessels by finding them very numerous and very large in the pulmonary veins of the cow, although on looking for them in swine, he found them absent.

In man, however, he found them, on examination, both large and numerous; so that it is difficult to understand how they should have escaped observation. A valve is always met with at the place where a venous branch joins the larger trunks at an acute angle; and the more acute this is, so much more marked is the valve. But where the branches join at a right angle no valve exists; which is precisely what takes place in the other parts of the venous system, as in the extremities, where valves exist where a branch joins the larger trunks at an acute, but not where this happens at a right angle. From this we see why it happens that fewer valves are met with in the pulmonary than in other veins; because the ramifications of the pulmonary veins chiefly take place at a right angle. This form of distribution occurs particularly in swine—and hence in their pulmonary veins there are no valves.—*Zeitschrift der Physiologie, Tom. III. p.* 155.

2. *Microscopic Researches upon the intimate structure of Animal Tissues.* By M. Raspail.—This memoir of a skilful and conscientious microscopic observer, is entirely opposed to the facts lately advanced by Messrs. Prevost, Dumas, Milne Edwards, &c. The following presents a rapid analysis.

1st. The membranes isolated and reduced to their essential consistence, are not composed of globules perceptible by our means of observation, and however coarse those examined may be, their surface will appear smooth and not granulated. If a filament be taken from a membrane, serving as a sheath to the muscular fasciculi, and placed in water, examination with the strongest microscope will only show a smooth surface, as transparent as the water itself. Some granulations of an irregular size and form, may be observed upon this surface, but these are evidently nothing more than little fatty follicles which do not properly belong to the membrane.

2d. *The Blood.*—The form and size of the globules differ in different men, as well as in various animals, and according to the vessels in which they have been found. They are smallest in the capillaries. The globules are vesicles filled with a substance probably albuminous. Those of the blood in nowise differ from those found in all the animal tissues.

3d. *Epidermis.*—The epidermis offers to the microscope the appearance of a bed of cells of greater or less thickness, flattish and in their outline irregular. Here and there, in their interior, granulations of various forms and sizes are perceptible, separated by spaces in which not the smallest globule is to be seen. The epidermis is nothing more than the external bed of the dermoid cells hardened by the air. The hairs are developed in the same manner as those of vegetables, from which they only differ by containing a fatty substance in their internal cells. The pits designated by Eichborn as the origin of the perspiratory canals, are to be seen, but the opening of these canals is not perceptible.

4th. *Nervous Tissue.*—If a filament from a nervous trunk, be examined by the microscope, the trunk is seen to consist only of an agglutination of cylinders, the fiftieth part of a millimetre in diameter. Fontana has proved that these cylinders are composed of a smooth and transparent membrane containing within "a glutinous, elastic and transparent matter, that does not dissolve in the water in which the cylinders float." Having pressed out this matter between two glasses, he caused it to return again by diminishing the pressure. M. Raspail considers each of these cylinders as a cellule which has grown only in length. It encloses a true cellular tissue, imbued with a homogeneous and fatty substance. It is without any longitudinal cavity. In its growth, it extends itself from the encephalon to the extreme ramifications.

5th. *The Muscular Tissue.*—The last analysis of this tissue, shows it to be composed of cylinders wound together spirally, and closely glued together. In the ox, the size of these cylinders is one twentieth of a millimeter. They are full like those of the nerves, from which, however, they differ by their rose colour. Their smooth sides exhibit cells on their interior, varying in form and size. Each fasciculus of cylinders is enveloped by a smooth sheath. Several of these bundles thus united together are again enclosed in a common sheath, and so on in succession.—*Journal des Progres, Vol. IX from the Repertoire d'Anatomie, &c.*

3. *Case of Disease of the Brain illustrating the Functions of the Fifth Pair of Nerves.* By E. Stanley, Esq.—A lady, aged forty, was attacked, immediately after her confinement, with fever and inflammation of the brain; after which, she suffered severe and almost constant pain in the head. Again becoming pregnant, she was confined about three months before her death. When nearly recovered from this confinement, she was attacked with pain in the head, more acute than usual, and delirium. These symptoms subsiding, hemiplegia supervened, and continued through the remaining two months of her life. During this period, the following circumstances were particularly noticed:—

The hemiplegia was on the left side. In the face, sensation and motion were completely lost. In the arm and in the leg sensation remained.

There were frequent attacks of erysipelas in the face, but confined to that side which was deprived of sensation and motion.

In the left ear hearing was completely lost.

In the left side of the tongue sensation was lost, but motion remained.

Whilst in the right nostril, the mucous membrane was pale, in the left nostril it was constantly of a deep red colour, and there were several discharges of blood from it.

In the left eye the vessels, first of the conjunctiva, then of the deeper membranes, became inordinately distended with blood. Opacity and ulceration of the cornea soon followed, with the escape of the aqueous humour, and complete disorganization of the globe.

Upon the subsidence of the delirium which preceded the hemiplegia, the intellect became clear, and remained so to the moment of death.

The medical superintendence of the preceding case was confided to Dr. P. M. Latham and to Mr. Eyles. I visited the patient in the latter part of her illness, and the opportunity was given me of examining the brain, which presented the following appearances:—

Effusion of transparent fluid into the cellular tissue of the pia mater, and into the ventricles, to the extent of about four ounces.

Enlargement of the tuber annulare, especially on its left side, and in a direction to compress the fifth and seventh nerves against the basis of the skull. A section of the tuber annulare discovered within it a tumour about the size of a walnut, occupying the whole of its left side, and extending into the left crus cerebelli. The consistence of the tumour was firm, its colour brown, and specks of blood were dispersed through it. From this morbid structure the fifth and seventh nerves were detached. When examined close to their respective foramina in the basis of the skull, these nerves presented no unusual appearance in size or texture.

The history of the foregoing case may be interesting to physiologists, as it records an instance of disease in that part of the brain whence the fifth and seventh nerves are detached, producing in the parts supplied by those nerves certain effects agreeing with the experiments of Magendie and others. Here, however, was an experiment of Nature's own making, free from the objections which may attach to experiments upon living animals.

In the case now related, the morbid changes in the eye, consequent on the disease, at the origin of the fifth nerve, were precisely the same as are reported to follow the division of this nerve in a living animal. The abolition of the functions of the fifth nerve in the human subject by disease, and in the brute by its division, was alike followed by inflammation, destructive of the eye; and in the case before us, the excitement of the blood-vessels in the parts deprived of sensation and of motion, was further manifested in the erysipelas of the paralysed cheek, and in the inordinate repletion and rupture of the blood-vessels in the nostril of the same side."—*London Medical Gazette, Vol. I. No.* 18, 1228.

4. *Case in which there was a diminution of Sensibility on one side, without loss of the power of Motion; and a loss of Muscular Power on the other side, without any diminution of Sensibility.* By H. Ley, M. D.—The following case is extremely interesting, as illustrating in a striking manner the distinct functions of different nerves, as recently pointed out by Mr. Charles Bell, and some of the continental physiologists.

Mrs. W. was delivered by a midwife at Kilburn. Her labour was easy, but followed by profuse hæmorrage upon the separation of the placenta, and after its exclusion from the uterus.

She revived from the state of exhaustion immediately consequent upon the loss of blood, but at the end of about three or four days became feverish, and complained of severe head-ache; for a week, however, she had no other assistance than that of the midwife.

At the end of this time, (about ten days after her delivery,) the head-ache continuing, and being now accompanied with some degree of 'numbness on one side,' I was requested to see her.

I found her labouring under severe head-ache, not confined to, but infinitely more violent upon one side than the other, and occupying the region of the temporal and occipital bones above the mastoid process, and attended with considereble pulsation.

Upon one side of the body there was such defective sensibility, without, however, corresponding diminution of power in the muscles of volition, that she could hold her child in the arm of that side, so long as her attention was directed to it; but if surrounding objects withdrew her from the notice of the state of the arm, the flexors gradually relaxed, and the child was in hazard of falling.

The breast, too, upon that side, partook of the insensibility, although the secretion of milk was as copious as in the other. She could see the child sucking and swallowing, but she had no conciousness from feeling that the child was so occupied; turgescence of that breast produced no suffering, and she was unconscious of what is termed the *draught* on this side, altough that sensation was strongly marked in the other breast.

Upon the opposite side of the body there was defective power of motion, without, however, any diminution of sensibility. The arm was incapable of supporting the child; the hand was powerless in its gripe; and the leg was moved with difficulty, and with the ordinary rotatory movement of a paralytic patient; but the power of sensation was so far from being impaired that she constantly complained of an uncomfortable sense of heat, a painful tingling, and more than the usual degree of uneasiness from pressure, or other modes of slight mechanical violence.

Medicinal agents, including blood-letting, general and local, blisters, purgatives, &c. directed, first by myself, afterwards by Dr. P. M. Latham, to whose care I directed her in the Middlesex Hospital, were of little avail, and she at length left the hospital scarcely, if at all, benefitted.

At the end of a few months she again proved pregnant. Her delivery, at the full time, was easy and unaccompanied with hæmorrhage, or other formidable occurrence, but at the expiration of about ten days she complained of numbness on both sides. Her articulation was indistinct; she became more and more insensible, and sunk completely comatose.

Upon examination of the body no positive disorganization of brain could be detected. The ventricles, however, contained more than usual serum; and there were found, more especially opposite to the original seat of pain, thickening, and increased vascularity of the membranes, with moderately firm adhesion in some parts; in others an apparently gelatinous, transparent, and colourless deposit interposed between them."—*Ibid, Vol. I. No.* 25, 1828.

5. *On the Effects of the Division, or Organic Lesion of the Fifth Pair of Nerves.*—It appears from the experiments of M. Magendie, H. Mayo, and C. Bell, on the action of the cerebral nerves, that on the division of the fifth pair, or when it is in a diseased state, the eye undergoes some peculiar morbid alterations. M. Magendie informs us, (Journ. de Physiol. IV,) that after the division of this nerve, the cornea becomes opaque, and that it, as well as the iris, begins to inflame and suppurate; an effusion of lymph takes place in the interior of the eye, and gradually the whole globe passes into ulceration. All these experiments, however, did not satisfy M. Magendie, and could not, in fact, lead to a clear result, as on dividing the nerve, the internal carotid was invariably wounded; he therefore, in more recent experiments, divided the nerve before it passes over the pars petrosa, and then obtained an effect somewhat different from that described before; the eye was much less altered, the inflammation occupied its upper portion only, and but a very small segment of the upper circumference of the cornea became opaque. It appears, then, that the fifth pair of nerves exercises a direct influence on the nutrition of the eye; the different results of the experiments are easily accounted for by the circumstance, that in the former experiments of M. Magendie, the ophthalmic artery was separated from the internal carotid, and that thus the nutrition of the eye necessarily became affected.

The following pathological fact, reported by M. Serres, confirms the experiments of M. Magendie. A young man was admitted in the Hospital de la Pitie, on account of epileptic attacks; at the same time a slight inflammation of the right eye was observed, the cornea was opaque, and the sight was to a considerable degree affected. All these symptoms gradually increased, till the sight was completely lost, and the right eye and eyelid, as well as the right side of the nose and tongue, were quite insensible. The patient died eleven months after admission, in a violent epileptic fit. On examination, the ganglion of the fifth pair was found enlarged, of a yellow colour, and very vascular; and on its exit from the pons varolii, the nerve was covered with a gelatinous mass.

Professor Mayer, of Bonn, (Journ. der Chirug. and Augenheilk. T. x.) has recently performed many experiments, from which it appears that not only the division of the fifth pair is followed by morbid changes of the eye, but that the same effects take place after wounds of the neck. From eighteen experiments on dogs, horses, and pigeons, he comes to the following result:—1. The division of the cervical portion of the sympathetic nerve was sometimes made without any effect on the nutrition of the eye; in other cases it was followed by redness and inflammation of the conjunctiva. 2. The same morbid change, in most cases, followed the division of the pneumogastric nerve. 3. The sympathetic and pneumogastric nerves having been divided, a very intense inflammation of the eye took place, which extended to its internal parts. 4. If the carotid was tied, and at the same time the nerves in its neighbourhood were carefully avoided, the nutrition of the eye was in no manner influenced. 5. After a ligature of both carotids, the eyes suffered more or less; they became dim and opaque, but very seldom a complete disorganization ensued. 6. But if the ligature comprised the pneumogastric or sympathetic nerve, an effusion took place from the enterior surface of the iris, the pupil was closed by a false membrane, and the cornea passed into suppuration.

6. *Vision after Destruction of the Optic Nerves.*—Magendie gives an instance of sight existing after the destruction of the optic nerves, in a man by name of Bardon, who was admitted into the Hotel Dieu, in 1827, and there died. On dissection, the following appearances presented themselves. In the interval between the crossing of the optic nerves and the pons varolii, was a cyst of the size of a small egg, this cyst was filled with a yellowish matter, of which one-third was solid, on the sides, and above, the cyst had flattened and almost destroyed the optic nerves, all that remained of them were portions of cerebral substance adhering to the cyst, and which were wholly deficient at the commissure; in fact, there was no connection between the eye and brain, except by the cyst. This patient could distinguish objects a few days before his death.—*Bull. Sci. Med. April*, 1828.

7. *On the effects of Galvanism on the Nerves.*—Of the numerous experiments which have been instituted, to verify the analogy between galvanism and the nervous action, those of Weinhold are not the least interesting or least curious. The following are the most remarkable.

He beheaded a cat, and after pulsation and muscular action had completely ceased, he removed the spinal marrow, and filled the vertebral canal with an amalgam of mercury, zinc and silver. Immediately the throbbing of the arteries recommenced, and the muscular actions were renewed, which it was impossible to distinguish from those which are produced by the influence of the spinal marrow; the animal made many leaps. When the irritability appeared exhausted Weinhold, by means of a metallic arc, placed the heart and voluntary muscles, gradually in contact with the artificial medullary substance, and he revived again general but feeble contractions.

He filled with the same amalgam, the cranium and vertebral canal of another cat, which did not give any sign of life; the animal became during about twenty minutes in such a state of vital tension, that it raised its head, opened its eyes, looked steadily, attempted to walk, and endeavoured to rise afrer falling down frequently. During all this time the circulation and pulsation were very active, and continued for a quarter of an hour after the chest and abdomen were opened. The secretion of the gastric juice was evidently more abundant than ordinary, and the animal heat was perfectly re-established.

Weinhold filled also the cranium only, of a dog with the same amalgam, he examined then the principal functions of the senses, and observed that the pupil still contracted, that the animal manifested still a desire to avoid the light, when a lighted candle was placed near it, and that it listened when a person struck with a key upon a table.

Weinhold remarked also that the two extremities, of a divided nerve, gave sparks when they were brought together. He divided the crural nerve in a cat, and placed the extremities at the distance of a line apart, and connected them by a metallic arc: the moment when he completed the chain, he saw at each extremity of the nerve a luminous point, but they did not pass from one to the other.

The hypothesis of nervous atmosphere has been completely subverted by the experiments of Weinhold, in which, after having cut the crural nerve, he could not excite the contraction of the thigh by means of galvanism, although the extremities of the nerves were at the distance of a line or even of a quarter of a line. The ligature, even of nerves, prevents the propagation of galvanism. He observed further that the nervous pulp is the only conductor of the galvanic action, whilst the neurilema is entirely deprived of this power.

Weinhold has observed even material changes which happen in the nervous system during the action of galvanism. Having isolated the crural nerve of a frog, he observed that the medulla of the nerve, which was almost transparent, retracted during the contraction of the muscles, produced by galvanic irritation, and that this retraction alternated with the dilatation. He laid bare the tracheal nerve of a rabbit, and he observed that after having produced twenty or thirty rapid contractions of the members, by means of a galvanic pile, the size of the nerve was diminished, lost its cylindrical form, and ultimately was reduced to a simple white and compresed tube. This loss of substance of the nervous medulla, during the action of nerves, was in the space of twenty or twenty-five minutes, repaired by the increase in the beating of the heart, coinciding with the violent contractions of the muscles; so that the nerve after a certain time, was restored to its cylindrical form. When on the other hand the heart is extirpated so that the reparation of the loss of nervous substance, cannot be effected by means of the circulation, the atrophied nerve does not regain its primitive form. Weinhold has observed the same loss of substance in the portion of the spinal marrow, where the nerves of the anterior extremities go off, when he made the muscles of these members contract by means of violent and continued action of the galvinic pile, directed to its nerves.—During the action of the nerve, not only the mass of the nervous substance diminishes, but also its consistence. When he divided a nerve, and irritated it a long time by means of galvanism, he observed that the nervous matter gradually became softer, and finally flowed guttatim, from the extremity of the divided trunk."—*Jour. des Progres, Vol. X.* 1828.

These are certainly marvellous experiments, and if their correctness should be confirmed by further observations, we need no longer despair of the Promethean art being attainable.

